# Activation of SAPK/JNK mediated the inhibition and reciprocal interaction of DNA methyltransferase 1 and EZH2 by ursolic acid in human lung cancer cells

**DOI:** 10.1186/s13046-015-0215-9

**Published:** 2015-09-11

**Authors:** Jingjing Wu, Shunyu Zhao, Qing Tang, Fang Zheng, YuQin Chen, LiJun Yang, Xiaobing Yang, Liuning Li, WanYin Wu, Swei Sunny Hann

**Affiliations:** Laboratory of Tumor Molecular Biology and Targeted Therapies, Guangdong Provincial Hospital of Chinese Medicine, The Second Clinical Medical Collage, University of Guangzhou Traditional Chinese Medicine, Guangzhou, 510120 Guangdong Province P. R. China; Department of Medical Oncology, Guangdong Provincial Hospital of Chinese Medicine, The Second Clinical Medical Collage, University of Guangzhou Traditional Chinese Medicine, Guangzhou, Guangdong Province 510120 P. R. China; No. 55, Neihuan West Road, Higher Education Mega Center, Panyu District, Guangzhou, Guangdong Province 510006 P. R. China

**Keywords:** Human lung cancer cells, SAPK/JNK, DNMT1, EZH2, Ursolic acid, SP1

## Abstract

**Background:**

Ursolic acid (UA), a pentacyclic triterpenoid, is known to have anti-tumor activity in various cancers including human non small cell lung cancer (NSCLC). However, the molecular mechanisms underlying the action of UA remain largely unknown.

**Methods:**

Cell viability was measured by MTT assays. Apoptosis was analyzed with Annexin V-FITC/PI Apoptosis Detection Kit by Flow cytometry. Western blot analysis was performed to measure the phosphorylation and protein expression of stress-activated protein kinase/c-Jun N-terminal kinase (SAPK/JNK), DNMT1 [DNA (cytosine-5)-methyltransferase 1], enhancer of zeste 2 polycomb repressive complex 2 subunit (EZH2) and SP1. Exogenous expression of SP1 and DNMT1 was carried out by transient transfection assays.

**Results:**

We showed that UA inhibited the growth and induced apoptosis of NSCLC cells in the dose- and time-dependent fashion. Furthermore, we found that UA induced phosphorylation of SAPK/JNK and suppressed the protein expression of DNMT1 and EZH2. The inhibitor of SAPK/JNK (SP600125) blocked the UA-reduced expression of DNMT1 and EZH2. In addition, UA suppressed the expression of SP1 protein. Conversely, overexpression of SP1 reversed the effect of UA on DNMT1 and EZH2 expression, and feedback attenuated UA-induced phosphorylation of SAPK/JNK. Moreover, exogenous expression of DNMT1 antagonized the effect of UA on SAPK/JNK signaling, EZH2 protein expression, and NSCLC cell growth.

**Conclusion:**

Our results show that UA inhibits growth of NSCLC cells through SAPK/JNK-mediated inhibition of SP1; this in turn results in inhibition the expression of DNMT1 and EZH2. Overexpression of DNMT1 diminishes UA-reduced EZH2 protein expression. The negative feedback regulation of SAPK/JNK signaling by SP1 and DNMT1, and the reciprocal interaction of EZH2 and DNMT1 contribute to the overall effects of UA. This study leads to important new insights into the mechanisms by which UA controls growth of NSCLC cells.

## Introduction

Lung cancer is the most common malignancy worldwide and a leading cause of cancer-related deaths. Despite recent progress in understanding the tumorgenesis signaling network and in producing new therapeutic strategies, this malignancy especially non-small cell lung cancer (NSCLC) still shows poor prognosis and high incidence of recurrence [1]. Majority of lung cancer presents at an advanced stage and treatment results in poor outcome [1, 2]. As a result, there is a significant interest in developing adjunctive therapeutics to augment available treatment regiments without compromising therapeutic efficacy. Ursolic acid (UA) may be one such potential candidate.

UA, a triterpene compound came from certain traditional medicinal plants [3], has been widely used for its anticancer properties via variety of biological functions involved in inducing cell apoptosis, anti-proliferation, chemo- and radiotherapy sensitization, anti-invasion and metastases [4–6]. In such, multiple signaling pathways and potential gene targets involved in UA-inhibited cancer cell growth including lung cancer have been reported in the past [5–8]. However, the molecular mechanisms underlying the beneficial effects of UA in the treatment of lung cancer remain largely unknown.

DNA methylation is controlled by DNA methyltransferase (DNMT), an enzyme that catalyzes the transfer of a methyl group to DNA. DNMT1 [DNA (cytosine-5-)-methyltransferase 1] is primarily a DNA methyltransferase that maintains DNA methylation, which is one of the regulatory mechanisms of gene expression [9]. DNMT1 also participates in several cellular functions other than through DNA methylation [10, 11]. Overexpression of DNMT1 has been found in various cancer types including lung cancer and inhibition of DNMT1 suppressed growth and induced apoptosis of cancer cells through multiple signaling pathways and distinct mechanisms [12–14]. Thus, re-expression of methylation-silenced tumor suppressor genes through inhibition of DNMT1 has emerged as an effective therapeutic strategy for cancer. Furthermore, study has focused on developing small DNMTs inhibitors, which can potentially be used as anti-tumor drugs [15].

Enhancer of zeste 2 polycomb repressive complex 2 subunit (EZH2), a polycomb group protein, has been identified as an oncogene in many types of cancers [16]. EZH2 is highly expressed in a various of human solid tumors and mediates the expression of several target genes that involved in tumorigenesis, cell cycle control, growth, and differentiation [17]. Overexpression of EZH2 is associated with tumor growth, metastasis and poor prognosis in multiple cancer types, including lung cancers [18–20]. Thus, targeting EZH2 may consider as a therapeutic potential for the treatment and prevention of lung malignancy [21, 22].

The association of UA effect and regulation of either DNMT1 or EZH2 expression have not been reported. In this study, we explored the potential mechanism by which UA inhibited growth of human lung cancer cells. We showed that UA suppressed growth and induced apoptosis of NSCLC cells through stress-activated protein kinase/c-Jun N-terminal kinase (SAPK/JNK)-mediated reduction of SP1 transcription factor; this in turn results in inhibition of DNMT1 and EZH2 expression.

## Materials and methods

### Reagents and cell culture

Monoclonal antibodies specific for total SAPK/JNK and the phosphor-form (thr183/tyr185) were purchased from Cell Signaling Technology Inc. (Beverly, MA, USA). The DNMT1 and EZH2 antibodies were obtained from Epitomics (Burlingame, CA, USA). SP600125 (JNK inhibitor) and antibody against SP1 were purchased from Merck Millipore (Millipore, Billerica, MA, USA). The 3-(4, 5-dimethylthiazol-2-yl)-2, 5-diphenyltetrazolium bromide (MTT) powder was purchased from Sigma Aldrich (St. Louis, MO, USA). Lipofectamine 3000 reagent was ordered from Life Technologies (AB & Invitrogen) (Carlsbad, CA, USA). Ursolic acid was purchased from Chengdu Must Bio-technology Company (Chengdu, Sichuan, China). The drugs were freshly diluted to the final concentration with culture medium before experiment. Human NSCLC cells (PC9, H1299, A549, H1650, H358 and H1975) were obtained from the Cell Line Bank at the Laboratory Animal Center of Sun Yat-sen University (Guangzhou, China) and the Chinese Academy of Sciences Cell Bank of Type Culture Collection (Shanghai, China). The cells were cultured at 37 °C in a humidified atmosphere containing 5 % CO2. The culture medium consisted of RPMI 1640 medium (Life Technologies, Carlsbad, CA, USA) supplemented with 10 % (v/v) heat-inactivated fetal bovine serum (Thermo Fisher Scientific Inc, Waltham, MA, USA), 100 μg/ml streptomycin and 100 U/mL penicillin. When cells reached 80 % confluence, they were digested with 0.25 % trypsin for passage for the following experiments.

### Cell viability assay

The MTT assay was used to determine the cell viability as reported previously [23]. Briefly, NSCLC cells were harvested, counted and seeded into a 96-well μL (1 × 10^4^ cells/well). The cells were treated with increasing concentrations of UA for up to 72 h. Afterwards, 10 μL MTT solution (5 g/L) was added to each well and NSCLC cells were incubated at 37 °C for an additional 4 h. After removing the supernatant, 150 μL solvent dimethyl sulfoxide (DMSO) was added to each well and oscillated for 10 min. Absorbance at 490 nm was determined through the use of ELISA reader (Perkin Elmer, Victor X5, Waltham, MA, USA). Cell viability (%) was calculated as (absorbance of test sample/absorbance of control) × 100 %.

### Cell apoptosis assays

Cell apoptosis was analyzed with Annexin V-FITC/PI Apoptosis Detection Kit (BD Biosciences, San Jose, CA, USA) according to instructions from the manufacturer. Briefly, after treated with UA for 24 h, the apoptotic cells were harvested by Trypsin without EDTA and washed with PBS. Afterwards, the cells were resuspended in 500 μL binding buffer, 5 μL Annexin V-FITC regent and 10 μL PI regents, and incubated for 5 min at room temperature (RT) in the dark, followed by detecting cell apoptosis by Flow cytometry (FC500, Beckman, USA).

### Detection of caspase 3/7 activity

We measured the activity of caspase-3/7 using the Caspase-Glo 3/7 Assay kit (Promega, Madison, WI, USA) according to the instruction from the manufacturer. Briefly, NSCLC cells were seeded in 96-well plates and treated with or without UA for 48 h. Afterwards, the cells were lysed and incubated with 100 μL of Apo-ONE Caspase-3/7 reagent (substrate and buffer in the ratio of 1:100). After 1 h incubation in the dark at RT, the fluorescence of each well was measured at 485–520 nm by reading in an Epoch Microplate Reader (Biotek Instruments; Winooski, VT, USA).

### Western blot analysis

The Western blot procedures were performed based on previous study [23]. Briefly, after determining the protein concentrations in cell lysates by Bio-Rad protein assay. The cell lysates containing equal concentration of protein were solubilized in 5x SDS-sample buffer and separated on 10 % SDS polyacrylamide gels. Membranes (Millipore, Billerica, MA, USA) were incubated with antibodies against SAPK/JNK, SP1, DNMT1 and EZH2 (1:1000). The membranes were then washed and incubated with a secondary antibody against rabbit IgG conjugated to horseradish peroxidase. The membranes were washed again and transferred to freshly made ECL solution (Immobilon Western; Millpore, Billerica, MA, USA), followed by observing the signals under the Molecular Imager ChemiDoc XRS Gel Imagine System (Bio-Rad, Hercules, CA, USA) and documenting the results.

### Electroporated transfection assays

The procedure was based on the protocol from the provider (Bio-Rad, Hercules, CA, USA) and reported previously [23]. Briefly, NSCLC cells (1 × 10^7^ cells/mL) were transferred into conical tubes and centrifuged at 1200 rpm for 5 min. After centrifuging, the medium were removed and the cells were washed with 1X PBS, and centrifuged again at 160 × g for 5 min. Afterwards, the PBS was aspirated and the tubes were added Bio-Rad Gene Pulser electroporation buffer. After resuspending the cells, the desired control (pCMV6) or expression constructs containing Myc/FLAG-tagged ORFs of human DNMT1and EZH2 were obtained from OriGene Technologies, Inc. (Rockville, MD, USA), or control (pcDNA3.1) and SP1 overexpression vector (pcDNA3.1SP1/flu, kindly provided by Dr. Thomas E. Eling (NIEHS, Research Triangle Park, NC, USA) [24] at a final concentration of 5–10 μg/mL were added, and the electroporation plate were put in the MXcell plate chamber in Gene Pulser II Electroporation System (Bio-Rad, Hercules, CA, USA). The electroporation conditions on the plates to deliver 150 V/5 ms square wave were adjusted. After electroporation was completed, the cells were transferred to a tissue culture plate. We typically transfer each 150–200 μL electroporation sample to a 6-well tissue culture plate containing 2 mL RPMI1640. Cells were incubated 48 h at 37 °C, then treated with UA for the indicated time for other experiments.

### Statistical analysis

All experiments were repeated a minimum of three times. All data are expressed as mean ± SD in triplicate measures. Differences between groups were assessed by one-way ANOVA and significance of difference between particular treatment groups was analyzed using Dunnett’s multiple comparison tests (GraphPadPrism5.0 software, LaJolla, CA, USA). Asterisks showed in the figures indicate significant differences of experimental groups in comparison with the corresponding control condition. *P*-values <0.05 were considered statistically significant.

## Results

### UA inhibited the growth and induced apoptosis of NSCLC cells in the dose-and time-dependent fashion

**Fig. 1:**
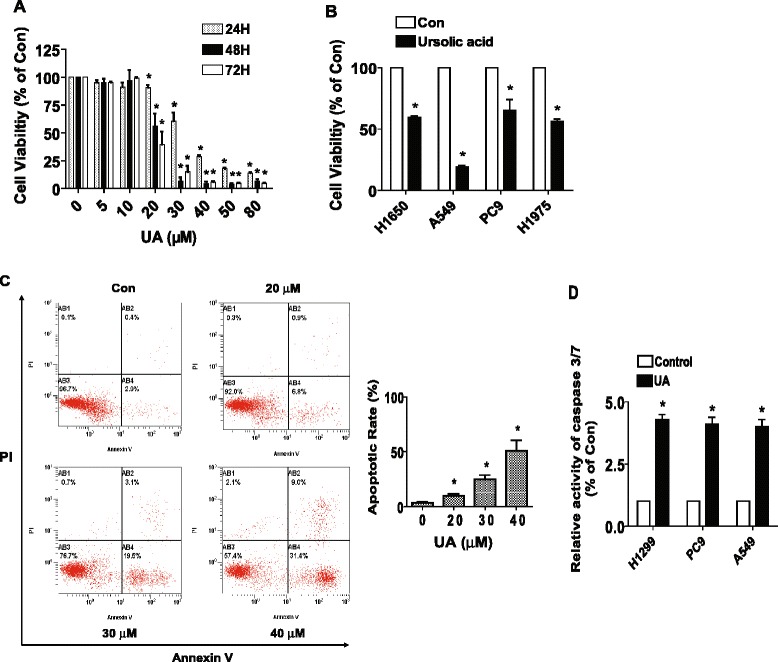
UA inhibited the growth and induced apoptosis of NSCLC cells in the dose-and time-dependent fashion. **a**, H1299 cells were treated with increased concentrations of UA for up to 72 h. **b**, NSCLC cell lines indicated were treated with UA (30 μM) for 48 h. The cell viability was determined using the MTT assay as described in the Materials and Methods Section and was expressed as percentage of control in the mean ± SD of three separate experiments. *Indicates significant difference as compared to the untreated control group (*P* < 0.05). **c**, H1299 cells were treated with increased concentrations of UA for 24 h. Afterwards, cells were harvested for analysis of apoptosis using the Annexin V-FITC/PI Apoptosis Detection Kit as detailed in Materials and Methods Section. The AB3 quardrant (annexin V-/PI-), AB4 quadrant (annexin V+/PI-) and AB2 quadrant (annexin V+/PI+) of the histograms indicated the percentage of non-apoptotic cells, early apoptosis and late apoptosis, respectively. Data are expressed as a percentage of total cells. Values in bar graphs were given as the mean ± SD from three independent experiments performed in triplicate. *indicates significant difference as compared to the untreated control group (*P* < 0.05). **d**, Caspase 3/7 activity assay was performed on H1299 cells treated with or without UA (30 μM) for 48 h. Relative caspase 3/7 activity is indicated as percentage of untreated control cells. Results represent those obtained in three experiments. *Indicates significant difference as compared to the untreated control group (*P* < 0.05)

We first detected the effect of UA on cell growth in human NSCLC cells H1299 by MTT assay. As show in Fig. 1a, UA decreased the cell viability in a dose- and time-dependent manner with maximal dose of 30 μM at 48 h treatment. Similar results were also observed in other NSCLC cell lines (Fig. 1b). The IC 50 was 20.71 μM (48 h).

Next, we examined the effect of UA on apoptosis in NSCLC cells. We found that H1299 cells treated with increased concentrations of UA for 24 h resulted in induction of apoptosis shown in the lower right (AB4) quadrants of the histograms, which were counted as “early” apoptotic cells (Fig. 1c) as detected by the Annexin V-FITC/PI stain Apoptotic Detection Kit. After 24 h of treatment, the UA-induced apoptosis rate was greater than that in the non-treated control cells (Fig. 1c). Meanwhile, the effect of UA on apoptosis of NSCLC cells was also tested by measuring caspase 3/7 activity. We observed the increased caspase 3/7 activity by UA in several NSCLC cell lines (Fig. 1d). Together, the results above suggested that UA inhibited growth and induced apoptosis in NSCLC cells.

### UA induced phosphorylation of SAPK/JNK

**Fig. 2:**
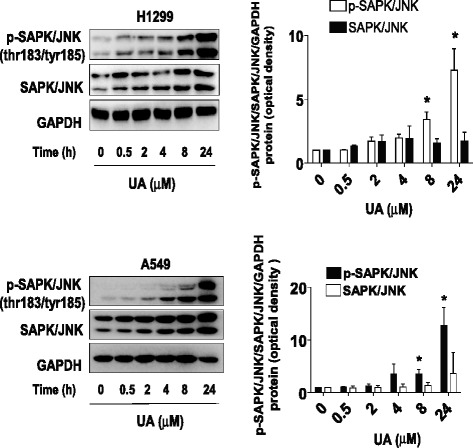
UA induced phosphorylation of SAPK/JNK. **a-b**, H1299 cells were treated with UA (30 μM) in the indicated times, and cell lysate was harvested and the expression of the phosphorylated and total protein of SAPK/JNK were measured by Western blot analysis using corresponding antibodies. GAPDH was used as loading control

Multiple growth factors and extracellular stimulus trigger activation of the mitogen-activated protein kinases (MAPK) signaling, such as SAPK/JNK pathway, this in turn lead to wide range of biological responses. The SAPK/JNK signaling pathway controls many facets of cellular function, such as apoptosis and cell growth depending on the cell context. We showed that UA increased the phosphorylation of SAPK/JNK in a time-dependent manner in H1299 and A549 cells (Fig. 2). Note that the expression of total SAPK/JNK protein had no significant changes after UA treatment (Fig. 2).

### UA suppressed the protein expression of DNMT1 and EZH2 in the dose-dependent manner through SAPK/JNK signaling pathway

**Fig. 3:**
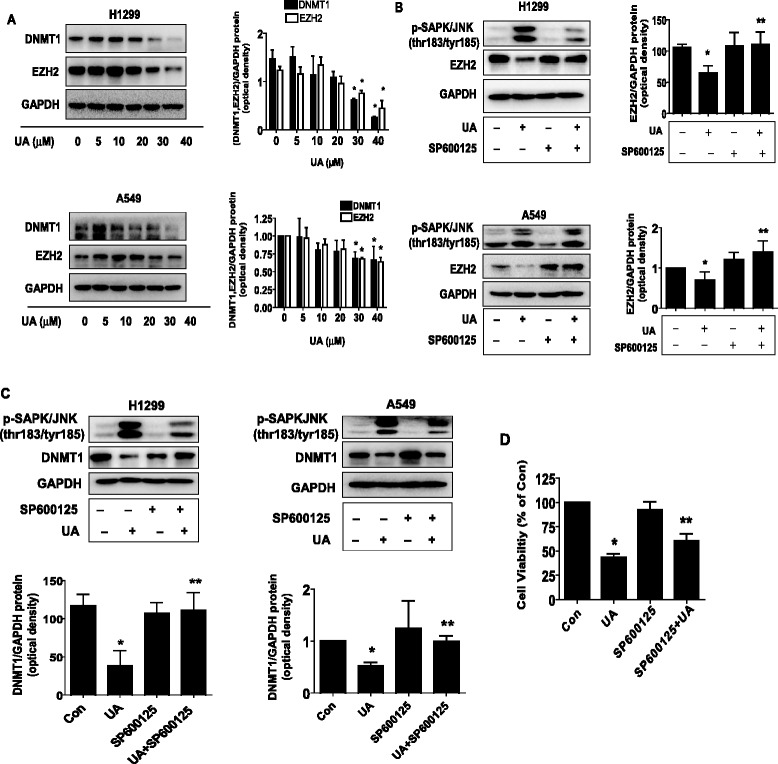
UA suppressed the protein expression of DNMT1 and EZH2 in the dose-dependent manner through SAPK/JNK signaling pathway. **a**, H1299 and A549 cells were exposed to increased concentration of UA for 24 h. **b**-**c**, H1299 and A549 cells were treated with SP600125 (20 μM) for 2 h before exposure of the cells to UA (30 μM) for an additional 24 h. Afterwards, the expression of EZH2 and DNMT1 protein were detected by Western blot using antibodies against EZH2 and DNMT1. The bar graphs represent the mean ± SD of EZH2 or DNMT1 /GAPDH of three independent experiments. **d**, H1299 cells were treated with SP600125 (20 μM) for 2 h before exposure of the cells to UA (30 μM) for an additional 24 h. Afterwards, The cell viability was determined using the MTT assay as described in the Materials and Methods Section and was expressed as percentage of control in the mean ± SD of three separate experiments. *Indicates significant difference as compared to the untreated control group (*P* < 0.05). **Indicates significant difference from UA treated alone (*P* < 0.05)

In this study, we showed that UA decreased protein expression of DNMT1, a DNA maintenance methyltransferase and EZH2, an oncogenic polycomb group protein [16], in a dose-dependent manner in H1299 and A549 cells (Fig. 3a). Next, we used special inhibitors of SAPK/JNK to pre-treated cells to examine the role of the kinase in mediating the effect of UA on reduction of DNMT1 and EZH2. As shown in Fig. 3b-c, we found that the special inhibitor of SAPK/JNK (SP600125) abrogated UA-reduced DNMT1 and EZH2 protein expression in H1299 and A549 cells. Note that SP600125 inhibited the phosphorylation of SAPK/JNK (Fig. 3b-c) and blocked the UA-inhibited cell growth (Fig. 3d). This result suggested that activation of SAPK/JNK was involved in the UA-reduced DNMT1 and EZH2 protein expression, and cell proliferation.

### UA suppressed the expression of SP1. Overexpression of SP1 attenuated UA -induced phosphorylation of SAPK/JNK, and –reduced DNMT1 and EZH2 protein expression

**Fig. 4:**
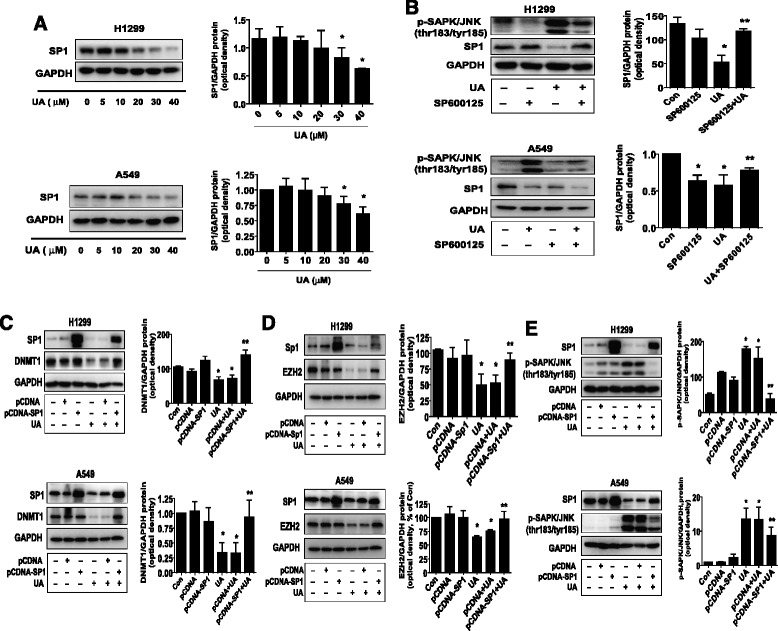
UA suppressed the expression of SP1. Overexpression of SP1 reversed the effect of UA on DNMT1 and EZH2 protein expression. **a**, H1299 and A549 cells were exposed to increased concentration of UA for 24 h, followed by measuring the protein expression of SP1 by Western blot. The bar graphs represent the mean ± SD of SP1/GAPDH of three independent experiments. **b**, H1299 and A549 cells were treated with SP600125 (20 μM) for 2 h before exposure of the cells to UA (30 μM) for an additional 24 h. Afterwards, the expression of phosphorylation of SAPK/JNK and SP1 protein were detected by Western blot. **c-e**, H1299 and A549 cells were transfected with control (pcDNA3.1) and SP1 overexpression vector (pcDNA3.1SP1/flu) for 24 h before exposing the cells to UA for an additional 24 h. Afterwards, SP1, EZH2 (**c**) and DNMT1 protein expression (**d**), and phosphorylation of JNK (**e**) were determined using Western blot. Values in bar graphs were given as the mean ± SD from three independent experiments performed in triplicate. *Indicates significant difference as compared to the untreated control group (*P* < 0.05). **Indicates significant difference from UA treated alone (*P* < 0.01)

Previous studies reported that DNMT1 gene promoter region contain SP1 binding sites and that SP1 regulated the expression and function of DNMT1 in several cell systems [25–27]. This was for this reason we tested the role of SP1. We showed that UA inhibited SP1 protein expression in the dose-dependent manner (Fig. 4a). Furthermore, a specific SAPK/JNK inhibitor (SP600125) abolished the effect of UA on SP1 expression in H1299 and A549 cells (Fig. 4b). Conversely, exogenous expression of SP1 transfected into the cells showed to resist the effect of UA on DNMT1 and EZH2 protein expression in H1299 and A549 cells (Fig. 4c-d). Interestingly, exogenous expression of SP1 reversed the effect of UA on phosphorylation of SAPK/JNK in H1299 and A549 cells (Fig. 4e), indicating a potential feedback regulation of SAPK/JNK signaling by SP1. Together, these results indicated the crucial role of SP1 in mediating the overall responses of UA in this process.

### Exogenous expression of DNMT1 overcame the effect of UA on EZH2 protein expression and cell growth

**Fig. 5:**
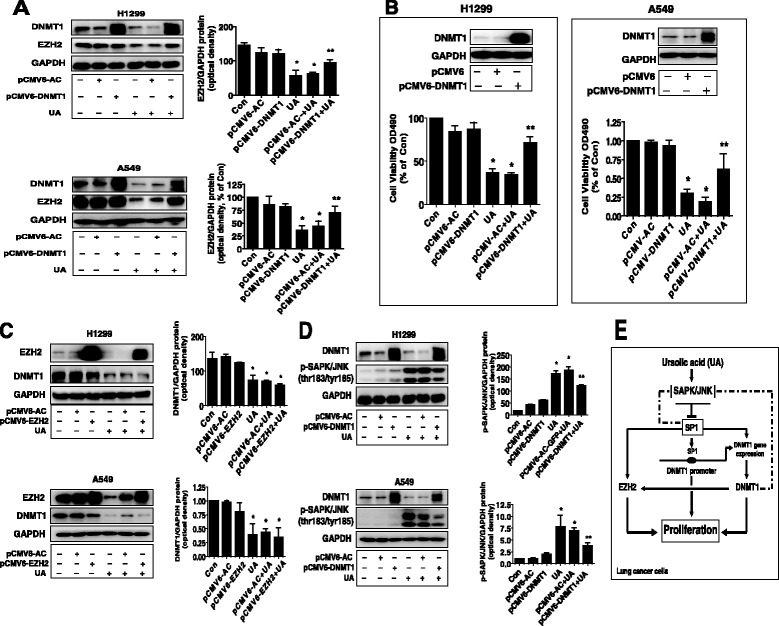
Exogenous expression of DNMT1 2 reversed the effect of UA on EZH protein expression and cell growth. **a-b**, H1299 and A549 cells were transfected with the control (pCMV6) or expression constructs of DNMT1 for 24 h before exposing the cells to UA for an additional 24 h. Afterwards, EZH2 and DNMT1 protein expression (**a**) and cell viability (**b**) were determined using Western blot and MTT assays. **c,** H1299 and A549 cells were transfected with the control (pCMV6) or expression constructs of EZH2 for 24 h before exposing the cells to UA for an additional 24 h. Afterwards, EZH2 and DNMT1 protein were determined using Western blot. **d**, H1299 and A549 cells were transfected with the control (pCMV6) or expression constructs of DNMT1 for 24 h before exposing the cells to UA for an additional 24 h. Afterwards, DNMT1, phosphor-SAPK/JNK were determined using Western blot. Values in bar graphs were given as the mean ± SD from three independent experiments performed in triplicate. *Indicates significant difference as compared to the untreated control group (*P* < 0.05). **Indicates significant difference from UA treated alone (*P* < 0.01). **e**, The diagram shows that UA inhibits NSCLC growth through SAPK/JNK-mediated inhibition of SP1; this in turn results in inhibition of DNMT1 and EZH2. Overexpression of DNMT1 diminishes UA-reduced EZH2 protein expression. The negative feedback regulation of SAPK/JNK signaling by SP1 and DNMT1 attenuates, while the reciprocal interaction of EZH2 and DNMT1 contributes the overall effect of UA in inhibition of lung cancer cell growth

Finally, we showed that exogenous expression of DNMT1 transfected into the cells reversed the effect of UA on EZH2 protein expression (Fig. 5a) and cell growth in H1299 and A549 cells (Fig. 5b), while overexpression of EZH2 had little effect on DNMT1 expression in H1299 and A549 cells (Fig. 5c). Intriguingly, overexpression of DNMT1 antagonized the effect of UA on SAPK/JNK signaling in H1299 and A549 cells (Fig. 5d). The above findings suggested that EZH2 could be an down stream of DNMT1 and that the reduction and interaction of EZH2 with DNMT1, and negative feedback regulation of SAPK/JNK signaling by DNMT1 contributed to the overall responses of UA.

## Discussion

Traditional Chinese medicine (TCM) has proved to be an important source for novel drug discovery with promising sources for a more effective and less toxic treatment option for cancer. There has been an increase attention on the study of TCM in the prevention and treatment of cancer including lung cancer [28, 29]. UA, one of naturally occurring pentacyclic triterpene acids, shows to have anti-growth activities against various cancers [4–6, 30]; however, a precise mechanism underlining this effect remains to be elucidated. In this study, we showed that UA inhibited the growth and induced apoptosis of NSCLC cells. The doses of UA used in this study were consistent with other studies, which showed significant growth inhibition in several cancer cell types [31–33].

Growing evidence also suggested that complicated signalling networks and multiple genes have been involved in the inhibition of cell growth and induction of apoptosis in NSCLC cells. Consistent with other reports [34, 35], we found that activation of SAPK/JNK, a family member of MAPK, mediated the effect of UA on inhibition of DNMT1, one of the DNA methyltransferases [36], EZH2, the core subunit of polycomb repressive complex 2 [16, 37], and growth of NSCLC cells. Of note, data from SAPK/JNK gene deletion in animal studies showed conflicting results suggesting the possible dual functions of this kinase (pro-oncogene or tumor suppressor) [38]. Therefore, the insight true role of SAPK/JNK in tumorgenesis and progression needs to be clarified in the future.

Our results demonstrated the involvement of DNMT1 and EZH2 in mediating the effect of UA on NSCLC cell growth inhibition. As crucial tumor promoters and oncogenes, EZH2 and DNMT1 are highly expressed in a wide range of cancer types and overexpression of EZH2 and DNMT1 are correlated with advanced stages of cancers, resulting in poor prognosis [13, 39]. Thus, deregulation or inactivation of EZH2 and DNMT1 could be important drivers of development, progression of tumor, and may be of therapeutic benefit for patients with malignancies including lung cancer [40, 41]. The interaction of DNMT1 and EZH2 has been shown in other studies [37, 42]. EZH2 regulated the activity of the DNMT1 through modulating DNA methylation, thus, served as a recruitment platform for DNA methyltransferases [37, 43]. The disruption of EZH2 and DNMT1 influenced tumorgenesis and cancer progression [44]. Interestingly, our results implied that DNMT1 is an upstream of EZH2, and that the potential interaction of DNMT1 and EZH2 contributed to the overall response of UA. One study showed that inhibition of DNMT1 not only decreased EZH2 binding to the promoter regions of cyclin-dependent kinases (CDK) inhibitors but also reduced EZH2 expression in human umbilical cord blood-derived multipotent stem cells (hUCB-MSCs) [45]. Nevertheless, additional experiments are required to better elucidate the true roles of these molecules involving in lung cancer cell survivals.

Intriguingly, our results also suggested the role of SAPK/JNK signaling pathway in mediating the effect of UA on inhibition of DNMT1 and EZH2 expression. Activation of SAPK/JNK associated with the regulation of EZH2 and DNMT1 expression has been shown in several other studies [46–48]. One report demonstrated that Epstein-Barr virus (EBV)-encoded latent membrane protein 1 (LMP1)-mediated activation and expression of DNMT1 involved in activation of JNK/MAPK in nasopharyngeal carcinoma cells [47]. SAPK/JNK is an upstream kinase involved in arsenic-stimulated phosphorylation of EZH2 in human lung epithelial cells [49]. Also, curcumin inhibited EZH2 expression through stimulation of MAPK pathway including SAPK/JNK in human breast cancer cells [50]. Collectively, our findings provided the insight into the connection between SAPK/JNK and regulation of DNMT1, EZH2 expression. The aforementioned also highlighted the tumor suppressor role of SAPK/JNK that mediated the anti-lung cancer effect of UA.

In this study, we revealed the role of SP1 involved in the UA-reduced DNMT1 and EZH2. SP1, a well-known ubiquitous transcription factor, is highly expressed in many types of malignancies and plays an important role in progression and metastasis of cancer through binding to GC-rich sequences, thereby regulating various downstream genes [51]. Targeting SP1 has been reported to have anti-tumor properties [52]. Our results demonstrated the pivotal role of SP1 in mediating the inhibitory effect of UA on EZH2 and DNMT1 expression. Consistent with this, one study showed that EZH2 was regulated by a novel signaling network including SP proteins in breast cancer cells and associated with poor survival of breast cancer patients [53]. Concomitant expression of DNMT1 and SP1, and the high GC sequence in proximal region of DNMT1 gene promoter indicated that DNMT1 could be a downstream target of SP1 [25, 27]. Thus, SP1 might control the expression of EZH2 and DNMT1 that converged to the UA-inhibited NSCLC cell growth, and thereby acting as a potential therapeutic target for the development of novel anticancer agents.

Interestingly, our results also demonstrated the negative feedback regulations of SAPK/JNK by SP1 and DNMT1, implying that the complicated regulatory loops were involved in the overall effects of UA. While limited data was available for the feedback links of SP1/DNMT1 to SAPK/JNK signaling pathway, the potential significance of these regulatory mechanisms involved in the overall responses of UA required to be determined.

We realized that less data were presented involving in the regulation of apoptotic signaling by UA in this study. It is possible that inhibition of proliferation can be in part a consequence of increased cell apoptosis or *vice versa.* Whether the SAPK/JNK /EZH2/DNMT1 signaling cascades also involved in the UA-induced apoptosis needs to be elucidated.

Collectively, our results show that UA inhibits NSCLC growth through SAPK/JNK-mediated inhibition of SP1; this in turn results in inhibition of EZH2 and DNMT1. Overexpression of DNMT1 diminishes UA-reduced EZH2 protein expression. The negative feedback regulation of SAPK/JNK signaling by SP1 and DNMT1 attenuates, while the reciprocal interaction of EZH2 and DNMT1 contributes to the overall effect of UA (Fig. 5e). This study leads to important new insights into the mechanisms by which UA controls growth of NSCLC cells and suggests that targeting of DNMT1 and EZH2 could be novel therapeutic potential for NSCLC prevention and treatment.

## Abbreviations

NSCLC: Non-small cell lung cancer
SAPK/JNK: Stress-activated protein kinases/c-Jun N-terminal kinases
DNMT1: [DNA (cytosine-5-)-methyltransferase 1]
EZH2: Enhancer of zeste 2 polycomb repressive complex 2 subunit
UA: Ursolic acid
MTT: 3-(4, 5-dimethylthiazol-2-yl)-2, 5-diphenyltetrazolium bromide
MAPK: Mitogen-activated protein kinase
EBV: Epstein-Barr virus
LMP1: Latent membrane protein 1
RT: Room temperature
CDK: Cyclin-dependent kinase
hUCB-MSCs: Human umbilical cord blood-derived multipotent stem cells
TCM: Traditional Chinese medicine
